# *Fasciola hepatica* hijacks host macrophage miRNA machinery to modulate early innate immune responses

**DOI:** 10.1038/s41598-021-86125-1

**Published:** 2021-03-24

**Authors:** Nham Tran, Alison Ricafrente, Joyce To, Maria Lund, Tania M. Marques, Margarida Gama-Carvalho, Krystyna Cwiklinski, John P. Dalton, Sheila Donnelly

**Affiliations:** 1 School of Biomedical Engineering, Faculty of Engineering and Information Technology, The University of Technology Sydney, Ultimo, NSW Australia; 2 School of Life Sciences, Faculty of Science, The University of Technology Sydney, Ultimo, NSW Australia; 3 BioISI–Biosystems & Integrative Sciences Institute, Faculty of Sciences, University of Lisbon, 1749-016 Lisbon, Portugal; 4 Center of One Health (COH) and Ryan Institute, School of Natural Science, National University of Ireland Galway, Galway, Ireland

**Keywords:** Immunology, Microbiology

## Abstract

*Fasciola hepatica*, a global worm parasite of humans and their livestock, regulates host innate immune responses within hours of infection. Host macrophages, essential to the first-line defence mechanisms, are quickly restricted in their ability to initiate a classic protective pro-inflammatory immune response. We found that macrophages from infected animals are enriched with parasite-derived micro(mi)RNAs. The most abundant of these miRNAs, *fhe-miR-125b*, is released by the parasite via exosomes and is homologous to a mammalian miRNA, *hsa-miR-125b*, that is known to regulate the activation of pro-inflammatory M1 macrophages. We show that the parasite *fhe-miR-125b* loads onto the mammalian Argonaut protein (Ago-2) within macrophages during infection and, therefore, propose that it mimics host *miR-125b* to negatively regulate the production of inflammatory cytokines. The hijacking of the miRNA machinery controlling innate cell function could be a fundamental mechanism by which worm parasites disarm the early immune responses of their host to ensure successful infection.

## Introduction

Helminths, or worms, are multicellular parasites that can live for many years within their vertebrate hosts. Of prime importance is the regulation of the host immune cell signalling pathways to prevent the parasite’s elimination before they can produce their off-spring in the form of eggs. Many worm parasites inactivate innate cell detection systems, such as macrophage and dendritic inflammatory responses, and direct the adaptive immune response towards a tolerant or hyporesponsive state^1–4^. Understanding how worm parasites do this will inform the design of new anti-helminthic treatments, such as drugs or vaccines.

*Fasciola hepatica*, or liver fluke, is a global zoonotic food-borne parasite that infects humans and their livestock. The parasite is one of the most successful helminths as it is found on every inhabited continent and infects the widest variety of mammalian hosts infected by any worm, including some that it has encountered only in relatively recent times (e.g. camelids and marsupials)^5,6^. This implies a superior adaptation to the mammalian host and suggests that *F. hepatica* parasites have evolved a universal process of invasion, tissue migration and immune modulation.

Mammalian hosts become infected with *F. hepatica* following ingestion of vegetation contaminated with encysted parasites (metacercariae). Within the intestine, juvenile parasites emerge from their cysts (termed newly excysted juveniles, NEJ) and within ~ 2 to 6 h traverse the intestinal wall and enter the peritoneal cavity. During the next 2–3 days of infection they rely on stored glycogen resources to migrate from the peritoneal cavity into the liver parenchyma where they begin to rapidly grow and develop. Their migratory activity in the liver causes the extensive haemorrhaging and tissue destruction associated with acute disease. During the invasion and pre-hepatic migratory stage, animals display no clinical signs of infection, and, histologically, no significant inflammatory changes are observed in the intestinal wall or peritoneal cavity^7,8^. This suggested that the parasites employ a mechanism(s) to disarm the host’s innate ‘early-response’ immune system to prevent their detection at a vulnerable time in their invasion.

Within the peritoneal cavity macrophages are the primary immune cell contributing to the immediate response to invading pathogens^9^ and are the predominant innate immune cell present during the first days of infection with *F. hepatica*^10,11^. Macrophages are major regulators of the inflammatory response as they monitor blood and tissues for signs of infection or damage and alert adaptive immune cells to any incursions. In response to invading pathogens, macrophages are typically ‘classically activated’ by signals from the pathogen itself and from damaged host tissue. This ‘M1’ phenotype of macrophage displays enhanced phagocytic and antigen presentation capacity and drives host protective immune responses by producing pro-inflammatory cytokines (such as TNF, IL-6 and IL-12) and anti-microbial effector molecules (like nitric oxide; NO)^12^. Despite the presence of migrating *F. hepatica* parasites, the levels of NO produced by peritoneal macrophages isolated from infected sheep are the same as macrophages from uninfected sheep^10,13^. Furthermore, peritoneal macrophages from infected sheep show no significant increase in the expression levels of pro-inflammatory cytokines (TNF, IL-12, IFNγ, iNOS)^14^. Importantly, this modulation of pro-inflammatory immune responses is also evident in experimental infections in mice^15–17^. The absence of a classical host protective inflammatory response to invading *F. hepatica* implies that the parasite possesses an effective mechanism of control on macrophage activity.

Helminth parasites secrete a range of soluble proteins/glycans that have been shown to suppress the ability of host macrophages to differentiate to M1 phenotype^18–20^. More recently, a role for miRNAs in the regulation of host immune responses by parasitic worms has been suggested^21–23^. Comparative analysis of parasite genomes has shown that some helminth miRNAs are widely conserved while others are specific to particular helminths^24,25^. Moreover, a number of the conserved miRNAs share sequence identity with mammalian miRNAs that are known to have an immune regulatory function. Target prediction software suggests that helminth miRNAs may act to regulate host immune responses to enable their development and survival^21–23^. However, to date, the evidence to support this proposed miRNA-mediated mechanism of immune modulation has come from in vitro analysis of transfected cells^26,27^ or the identification of parasite miRNAs in sera or tissue of infected hosts^28–30^. Here we show that *F. hepatica* releases miRNAs, that are homologous to host miRNAs, that regulate macrophage function in vivo and propose that worms may hijack this process to control their host’s early immune responses.

## Results

### Parasite-derived miRNAs are located within macrophages during infection with *Fasciola hepatica*

**Figure 1:**
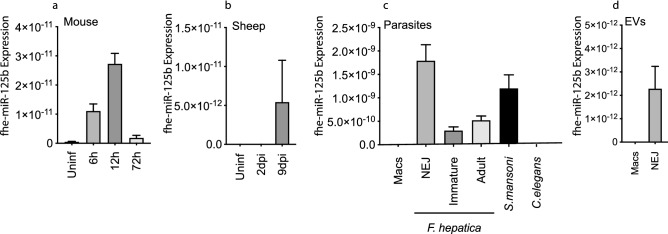
Worm-derived miRNA *fhe-miR125b* is detected within the peritoneal macrophages of mice and sheep infected with *Fasciola hepatica*. The presence of worm *fhe-miR125b* was (**a**) validated in the peritoneal macrophages that had been harvested from mice at 0 h, 6 h, 12 h and 72 h after an oral infection with 20 *F. hepatica* metacercariae and subjected to small RNA sequence analysis; and examined in (**b**) peritoneal cells harvested from uninfected sheep and from sheep at 2 and 9 days after an oral infection with 150 metacercariae. (**c**) The expression of *fhe-miR125b* was measured in RNA extracted from the *F. hepatica* newly excysted juveniles (NEJ), 21-day old worms (immature) and adult worms and compared to expression levels in adult (male and female mixed population) *Schistosoma mansoni* and *Caenorhabditis elegans.* (**d**) RT-qPCR using primers specific for *fhe-miR125b* was carried out on RNA extracted from NEJ extracellular vesicles (EVs). In these experiments, levels of *fhe-miR125b* expression determined by RT-qPCR are represented as the starting quantity of genetic material prior to amplification (N0) as determined by LinRegPCR (v.11). The data is presented as the mean ± SD of triplicate biological samples.

To investigate whether miRNAs produced by *F. hepatica* contributed to the inhibition of macrophage activation in early infection, we orally infected BALB/c mice with *F. hepatica* metacercariae and searched for parasite miRNAs in peritoneal macrophages at selected time-points (6 h, 12 h, 24 h, 3 days and 5 days after infection) using RNASeq (deposited in NCBI's GeneExpression Omnibus; accession number GSE145597; https://www.ncbi.nlm.nih.gov/geo/query/acc.cgi?acc=GSE145597). After first cleaning the datasets obtained, reads were mapped against the mouse database from miRBase (mmu). Then, to identify *F. hepatica-*specific miRNAs the remaining 15,796,193 reads were screened against the miRBase list of 30 *F. hepatica* miRNAs^31^. Using this approach, we identified 10 *F. hepatica* miRNAs that were present within peritoneal macrophages of infected mice (Table 1). *F. hepatica miR-125b (fhe-miR-125b*) was the most abundant miRNA observed in peritoneal macrophages during the first 24 h of infection, with expression peaking at approximately 12 h after infection. This is coincident with the migration of NEJs through the intestine and into the peritoneal cavity, and also with the observed early suppression of host innate immune responses, as described above^10,13,14^. This rapid and transient expression pattern of *fhe-miR-125b* in peritoneal macrophages was corroborated by RT-qPCR (Fig. 1a). Consistent with these observations, the expression of *fhe-miR-125b* was also observed in peritoneal cells isolated from sheep after an experimental infection with 150 metacercariae (Fig. 1b). These miRNAs were not detected in peritoneal macrophages taken from non-infected mice or sheep (Fig. 1a,b).

**Table 1:** Read numbers of known *Fasciola hepatica* miRNAs detected by miRNASeq in peritoneal macrophages harvested from infected mice over a time-course of 5 days.

*Fasciola* miR	Time after infection											
	Uninfect^a^		6 h		12 h		24 h		3 days		5 days	
*fhe-bantam*	–^b^	–	2	1	–	–	1	–	–	–	–	–
*fhe-miR-125b*	–	–	1	16	6	16	1	2	–	1	–	–
*fhe-miR-36a*	–	–	1	–	–	–	–	–	–	–	–	–
*fhe-miR-61*	–	–	–	3	1	5	–	3	–	–	–	–
*fhe-miR-125a*	–	–	–	1	–	–	–	–	–	–	–	–
*fhe-miR-46*	–	–	–	1	–	1	–	–	–	–	–	–
*fhe-miR-277*	–	–	–	2	–	3	–	–	–	–	–	–
*fhe-miR-10*	–	–	–	1	–	–	–	–	–	–	–	–
*fhe-miR-219*	–	–	–	–	1	1	–	–	–	–	–	–
*fhe-miR-71b*	–	–	–	–	–	1	–	–	–	–	–	–

^a^Two biological samples were sequenced at each time point with read counts for each sample presented in columns below.

^b^“–“ no read counts detected.

To verify that the primers were specifically detecting a miRNA of parasite origin, infectious metacercaraie were experimentally excysted in vitro and the RNA of the emerging newly excysted juvenile (NEJ) subjected to RT-qPCR. This confirmed that *fhe-miR-125b* is expressed by the NEJ parasites (Fig. 1c). RT-qPCR also demonstrated that *miR-125b* could be amplified from RNA isolated from *Schistosoma mansoni*, a trematode parasite like *F. hepatica*, but not from the free-living nematode *Caenorhabditis elegans*. This result concurs with reports demonstrating that the expression of a *miR-125b* is specific to trematode parasites^32^. Comparison of the expression of *fhe-miR-125b* across the different intra-mammalian life-stages of *F. hepatica* revealed strict temporal expression, supporting the idea that worm miRNAs govern developmental processes. In the context of a role in the immune modulation of innate immune responses after infection, it was notable that the expression of *fhe-miR-125b* was most abundant in NEJs, the stage that initiate infection.

Several studies suggest that extracellular vesicles (EVs) are the main reservoir of exogenous miRNAs. In this study, EVs were harvested from in vitro cultured NEJs and shown to contain *fhe-miR-125b* (Fig. 1d)*.* Since EVs released during helminth infections are primarily taken up by macrophages^26,27^ this provides a mechanism by which *fhe-miR-125b* and other miRNAs are delivered into the macrophages.

### *Fasciola hepatica miR-125b* targets innate immune signalling pathways

**Figure 2:**
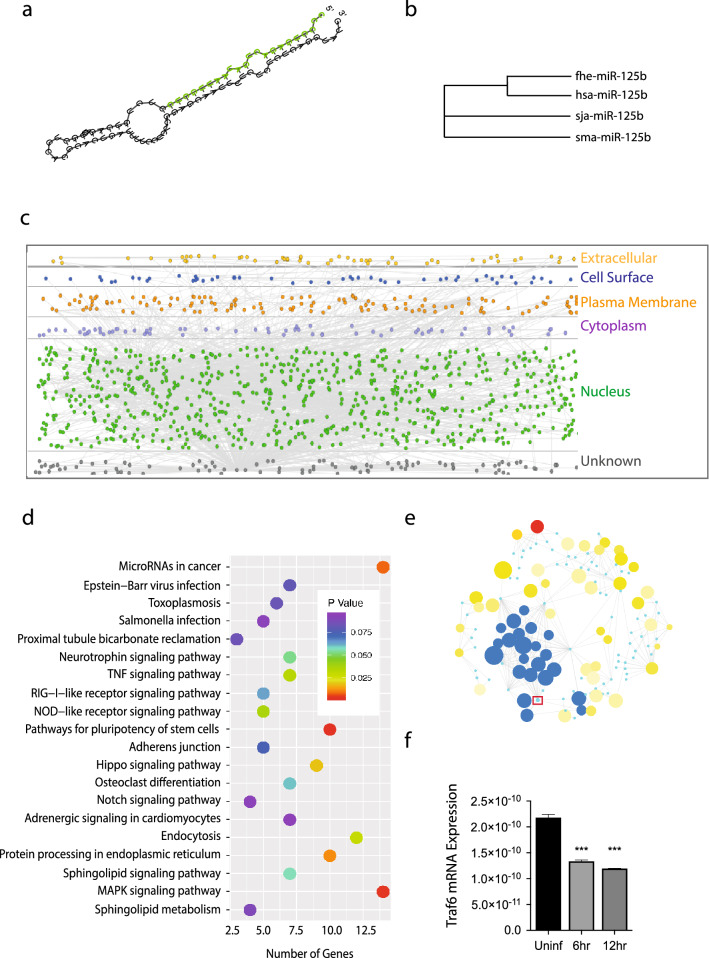
TRAF6 is the primary gene predicted as a target for *fhe-miR125b* in peritoneal macrophages*.* (**a**) The hairpin structure of *fhe-miR125b,* produced by RNAfold (http://rna.tbi.univie.ac.at/cgi-bin/RNAWebSuite/RNAfold.cgi) (**b**) Phylogram of miR125 generated using T-Coffee (http://tcoffee.crg.cat), based on the hairpin structures. (**c**) Illustration of a Protein–Protein Interaction map for the 798 immune-related genes predicted from gene target analysis (created by Innate DB; https://www.innatedb.com). Of these, the nucleus showed the highest number of interactions account for greater than 50% of all the interactions. (**d**) GO analysis of pathways represented by the predicted gene targets for *fhe-miR-125b,* produced using bioconductor (https://www.bioconductor.org)*.* (**e**) Mapping predicted gene targets to a mouse interactome produced 66 nodes (image created by Innate DB; https://www.innatedb.com). Within these, Traf6 (red box) mapped to 35–40% of the nodes (Blue dots). All other nodes not connected to Traf6 are shown in yellow and red. A detailed map of these interactions can be viewed in supplementary Fig. 3. (**f**) The expression of TRAF6 was measured in RNA extracted from peritoneal macrophages harvested from uninfected mice (Uninf) or from mice at 6 h and 12 h after an oral infection with *F. hepatica*. The level of expression is represented as the starting quantity of genetic material prior to amplification (N0) as determined by LinRegPCR (v.11). The data is representative of three independent experiments and is presented as the mean ± SD of triplicate biological samples. The data was analyzed using one-way analysis of variance (ANOVA).

The secondary structure of *fhe-miR-125b* indicates a typical miRNA with the mature strand located on the 5′ arm of the precursor (Fig. 2a). Previous studies have documented human *hsa-miR-125b* as a miRNA that regulates the inflammatory response of macrophages by controlling the activation of M1 macrophages^33–35^. Using a phylogenetic approach, we compared the structure of *fhe-miR-125b* to its human homologue and to miR-125 sequences from the closely related trematodes, *Schistosoma japonicum* and *mansoni* (Fig. 2b). This analysis revealed that miR-125b in Fasciola is closely related to the human miRNA, but is distinct from miR-125b found in the Schistosome parasites. This may suggest *fhe-miR-125b* could target the same host genes as *hsa-miR-125b* and thus play a significant role in the control of host immune responses during early infection.

To address this possibility experimentally, a list of putative cellular targets for mature *fhe-miR-125b*, (CCCCUGAGACUGAUAAUUGCUC) was initially generated using the online miRNA target prediction tool miRDB (https://mirdb.org). Only those genes assigned a target score > 60 (a predictive score generated by MirTarget^36^) were regarded as authentic and selected for further analysis. The resultant 511 genes (Supplementary Table 1) were mapped for protein–protein interactions using the innate immune data reference set^37^ and provided 798 immune-related protein–protein interactions with an enrichment in the nucleus (Fig. 2c). This dataset was then filtered down to cell specific associations restricted to peritoneal macrophages. Surprisingly, from this analysis, only two gene targets emerged; Traf6 and Nlrc5. Pathway analysis of the interactions showed that Traf6-mediated events were over-represented (Fig. 2e). To further scrutinise if Traf6 could be the prime target candidate for *fhe-miR-125b,* two additional predictive tools were then used, miRanda and TargetScan (Supplementary Table 1). Supporting the miRDB initial analysis, TRAF6 was one of only 43 genes that were commonly predicted as targets for *fhe-miR-125b* by all three tools. Furthermore, the significant reduction in the expression of TRAF6 in peritoneal macrophages harvested from mice at the same timepoints that *fhe-miR-125b* is most abundant (Fig. 2f), provides experimental evidence to validate the predictive analysis. As a transcription factor that is central to a number of immune signalling pathways, TRAF6 is regarded as an indispensable regulator of inflammatory responses^38^. Indeed, gene ontology analysis of the predicted gene target list conducted against a *Mus musculus* background using GO tool DAVID (http://david-d.ncifcrf.org) identified major signalling pathways known to control innate inflammatory responses (MAPK signalling, TNF signalling, NOD-like receptor signalling) within the top ten generated target KEGG pathways^39^ (Fig. 2d and Supplementary Table 2) thus providing more evidence for a role for *fhe-miR-125b* in the regulation of immune responses.

A central role for *fhe-miR-125b* in the regulation of these signalling pathways was also corroborated by expression profiling of macrophages harvested from the peritoneal cavity of mice 18 h after an oral infection with *F. hepatica*. Of the 610 genes that were identified to be significantly (p > 0.05) downregulated (> twofold change in expression), a large number of these were pro-inflammatory/innate cytokines and chemokines or transcription factors that regulate cytokine-signalling pathways (Supplementary Table 3). This is illustrated in the global KEGG analysis of these genes which created a profile suggesting involvement predominantly in MAPK, JAK-STAT, TNF and TLR signalling (Supplementary Fig. 1) and which was almost identical to that predicted for *fhe-miR125b* using GO tool DAVID above (Fig. 2).

**Figure 3:**
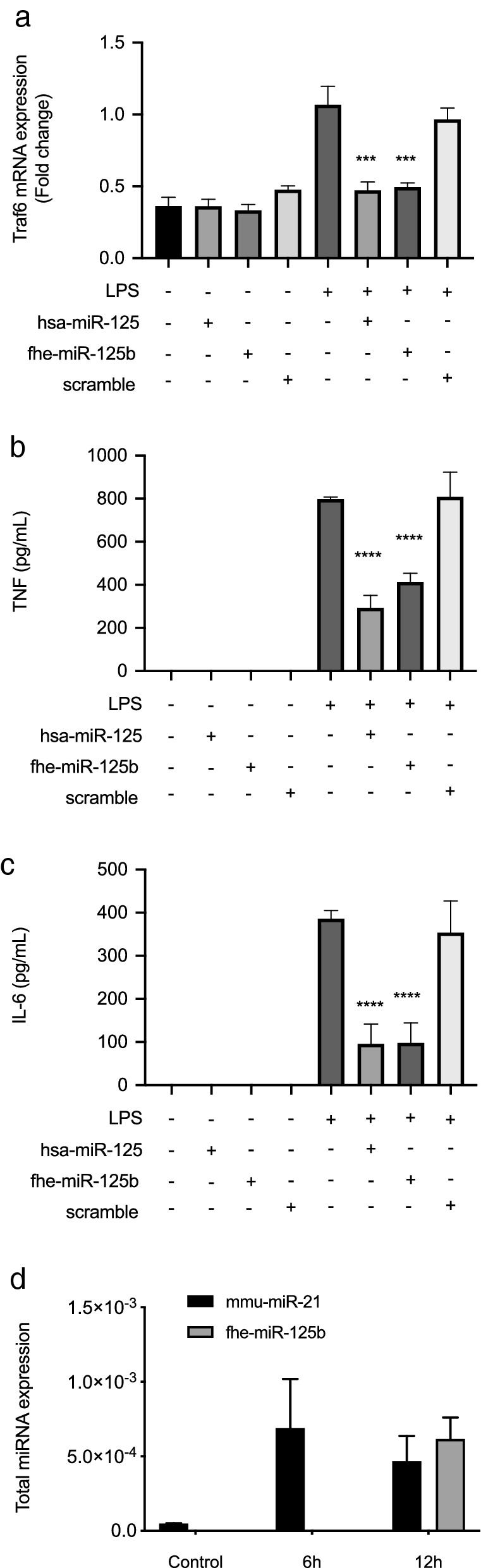
TRAF6 signaling and downstream production of IL-6 and TNF by macrophages is suppressed by *fhe-miR-125b* by binding the mammalian argonaute protein-2. Bone marrow derived macrophages were transfected with the parasite-derived *fhe-miR-125b*, the human *miR-125b* or a scrambled miRNA (20 nM) and stimulated 24 h later with bacterial lipopolysaccharide (LPS; 10 ng/ml) for 6 h. (**a**) The expression of TRAF6 was measured using RT-PCR and is presented the fold change in expression as compared to the LPS stimulated cells. The data is the mean ± SD of three independent experiments, each with biological triplicate samples. (**b**) The quantity of TNF and (**c**) IL-6 secreted by the macrophages into culture medium was measured by ELISA. The data shown is the mean ± SD of two independent experiments, each with biological triplicate samples. (**d**) Argonaute(Ago)-2 proteins were pulled down from extracted murine peritoneal macrophages pooled from five uninfected BALB/c mice (n = 3 pooled samples) and from five mice (n = 3 pooled samples) at 6 h and 12 h post-infection with *F. hepatica*. Ago-2-bound RNA was extracted and analysed using RT-qPCR with primers specific for *fhe-miR-125b*. The quantity of miRNA detected is presented as the starting quantity of genetic material prior to amplification (N0) as determined by LinRegPCR (v.11). Statistical significance was determined by one-way analysis of variance (ANOVA) with Tukey post hoc analysis.

To provide experimental proof of the ability of *fhe-miR-125b* to directly engage in the regulation of pro-inflammatory pathways in macrophages, primary murine macrophages were transfected with synthetic versions of both the *F. hepatica* and human *miR-125b*. While neither miRNA had any apparent effect on macrophages when transfected under basal culturing conditions, both transfections significantly inhibited the ability of macrophages to express TRAF6 in response to a challenge with bacterial LPS (Fig. 3a) and, as a consequence, the production of both IL-6 and TNF were significantly reduced (Fig. 3b,c). This outcome was specific to the miR-125b as transfection with a scrambled non-targeting miRNA had no effect. We propose, therefore, that the *F. hepatica fhe-miR-125b*, like its mammalian counterpart, functions to regulate the ability of macrophages to respond to pro-inflammatory signalling, thus preventing the differentiation to an M1 phenotype.

### *Fasciola hepatica miR-125b* complexes with the mammalian Argonaute-2 protein in host macrophages to regulate mammalian gene expression

The mechanism(s) by which the exogenous worm miRNAs operate in mammalian immune cells to alter their function remains enigmatic. However, studies in mammalian cells have shown that in the first step miRNAs must form a complex with an Argonaute protein (Ago). This Ago-miRNA association forms the core of the miRNA-induced silencing complex (miRISC), which is then guided by the complexed miRNA to bind complementary (target) sequences in the messenger RNA (mRNA)^40^. Helminth Ago proteins have been detected within secreted EVs and may be involved in the regulation of mammalian target genes^26^. However, given the similarity of *fhe-miR-125b* to its mammalian homolog we surmised that helminth miRNAs delivered into immune cells could directly load onto mammalian Ago2. In this way worm miRNAs would compete with the mammalian miRNA machinery to influence host gene expression. To test this idea, murine Ago2 was isolated by immunoprecipitation from the peritoneal macrophages taken from infected mice at 6 h and 12 h post infection (IP; Supplementary Fig. 2) and the miRNA extracted and subjected to RT-qPCR. We used the detection of murine miR-21 as a control for the pulldown and to demonstrate the normalisation of input miRNA to the RT-qPCR. This miRNA has been characterised as a central regulator of macrophage activity and its expression is enhanced by inflammatory stimuli^41–43^. Therefore, the observation of this murine miRNA loaded onto the Ago-2 within peritoneal macrophages of infected mice at both 6 h and 12 h, but not in uninfected animals (Fig. 3d) supports the efficacy of the pulldowns and validates the normalisation of miRNA input to the RT-qPCR. In contrast, the parasite-derived mir-125 was loaded onto Ago-2 only at 12 h timepoint (a time when *fhe-miR-125b* expression was at its peak; Table 1) reflecting the changes to the loading of mir-125b as a result of the progression of parasite infection (Fig. 3d).

## Discussion

On invasion of the host by the parasite *F. hepatica* protective pro-inflammatory cytokine expression in macrophages is quickly blocked. Thus, a major player in the innate cell immune system is inactivated at a stage when the parasite is most vulnerable and depends on its glycogen stores for energy to power its way from the intestine to the liver. Our study suggests that this trematode parasite, and perhaps others, exploit homologs of the immune-regulatory miRNAs of the host to hijack macrophage signalling pathways to disarm their function.

Several parasitic worm miRNAs share homology with mammalian miRNAs. Thus, they have the capacity to regulate the expression of an array of host genes within macrophages and other innate cells to effectively evade expulsion and continue their growth and development to maturity. Our gene target analysis and in vitro studies pinpoint a specific mechanism whereby *F. hepatica* could interrupt the MAPK signalling pathway of innate immune responses via the regulation of TRAF6 by *fhe*-*miR-125b*. This mechanism is supported by the presence of a single *miR-125b* binding site on the 3′ UTR of TRAF6^44^. The identification of mammalian genes and immune pathways targeted by parasite miRNAs has major implications for our understanding of the interaction between parasite and host.

It is highly significant that *miR-125b*, the most predominant parasite miRNA found in host macrophages of mice infected with *F. hepatica*, is conserved amongst the trematode clade of helminths, including the blood flukes of the genus *Schistosoma*, as it points towards a common mechanism for the anti-inflammatory control amongst these important parasites of humans and their livestock. Indeed, recent studies have shown that the *miR-125b* orthologue is highly enriched in EVs released by *S. mansoni* and *S. japonicum*, representing 40% and 65% respectively of the total miRNA reads^27,45^. However, in contrast to the immune modulatory effect of *fhe*-*miR-125b* we have shown here, transfection of murine macrophages with *sja-miR-125b* resulted in the induction of pro-inflammatory immune responses as assessed by an increased production of TNF^27^. Predictive analysis of this parasite miRNA led Liu et al.^27^ to suggest that the *sja-miR-125b* was targeting the expression of *Pros1,* a gene known to inhibit TLR-driven pro-inflammatory responses. Neither of the three predictive tools used in our study, predicted this gene as a target for the *F. hepatica miR-125b,* nor was it identified amongst the genes down-regulated in macrophages isolated from infected mice. These contrasting observations could suggest that *miR-125b* plays different functions for different parasites. Unlike *F. hepatica*, a pro-inflammatory Th1-type immune response dominates during the first 3–5 weeks of infection with Schistosome parasites. As the parasites mature and females become fecund, parasite eggs trapped in tissues induce potent Th2 responses that suppress Th1 responses^46^. The *sja-miR-125b* was identified within EVs isolated from parasites collected from mice at 28 days post-infection^27^, a pre-patent time point, coincident with a strong Th1 pro-inflammatory immune response.

A single miRNA can influence the expression of a multitude of different genes. mRNA silencing efficiency is determined by the sequence of the seed region, free energy produced by the miRNA-target duplex and stability of the miRNA 5′ terminal and seed region^47^. While high specificity of the miRNA seed sequence has been largely reported as the crucial factor to miRNA target recognition, nucleotide pairings outside of the seed are also known to affect gene target recognition and overall thermodynamic stability of the miRNA-target duplex^48^. These key concepts are integrated in predictive tools such as miRDB and MiRanda that scrutinise whole mature miRNA sequences. Examining the overall structure of miR-125 from various species, phylogenetic analysis indicates that *fhe-miR-125b* is closely related to its human counterpart and distal to that of miR-125b found in Schistosome. This may explain why *Pros1* was not predicted to be a target for *fhe*-*miR-125b* and only regulated by *sja-miR-125b*. It would be interesting to determine which exact nucleotides outside the seed region may account for this specificity.

miRNAs are not functional until they are loaded onto an Ago protein, a process that facilitates base-pairing to mRNA targets and ultimately translational repression and mRNA degradation. Here we provide proof that exogenous, worm-derived miRNA is delivered into macrophages, probably via exosomes, during infection and loads onto host Ago2. Coupled with our in vitro transfection studies that shows *fhe*-*miR-125b* can alter macrophage phenotype we can deduce that parasite miRNAs are functional within mammalian cells. We have to assume that this binding mimics that of host *hsa-miR-125b* and, therefore, operates in the same way, to regulate the pro-inflammatory response of macrophages^33–35^.

It has been reported that miRNAs that have homologs in mammalian hosts dominate the miRNA cargo *of F. hepatica* EVs. However, a number of miRNAs which are novel to the parasite have also been identified here and elsewhere^23^. Predictive analysis suggests that these too have the capacity to regulate immune-related mammalian genes^23,25^. As the loading of miRNAs to Ago protein is a very precise process with the requisite thermodynamics and base-match composition, it remains to be determined whether homology to a mammalian miRNA is essential for a parasite miRNA to associate with mammalian Ago and annex the mammalian miRNA machinery.

The ability of *F. hepatica* to invade, infect and produce progeny in a very broad range of species indicates a strong universal compatibility between the parasite and its mammalian host, particularly in terms of attuning the immune response. This study provides direct in vitro and in vivo evidence for the role of helminth-derived miRNAs in this complex parasite-host immune-interplay. Further elucidation of how mechanisms of immune modulation, involving nucleic acids, proteins and glycans, are employed by the parasite, especially during the early stage of infection, is critical for development of effective anti-helminth therapies to prevent infection and pathogenesis.

## Methods

### Murine and sheep infections

Six to eight week old female BALB/c mice (Australian Resource Centre, Perth, Australia) were orally infected with 20 metacercariae of *F. hepatica* (Baldwin Aquatics Inc, USA). Peritoneal exudate cells were collected by washing the peritoneal cavity with 5 ml of sterile saline. Ethical approval for this study was granted by the University of Technology Sydney (UTS) Animal Care and Ethics Committee (Approval Number: 2012-080) and experiments were conducted in accordance with the approved guidelines to be compliant with The Australian Code for the Care and Use of Animals for Scientific Purposes.

Ten 6 month-old male Dorset cross sheep (UK) were orally infected with 150 *F. hepatica* metacercariae (South Gloucester isolate, Ridgeway Research Ltd) administered in water. Animals were euthanized at selected time points post infection by captive bolt and peritoneal cells harvested as previously described^14^. These experimental procedures were reviewed and approved by the Agri-Food Biosciences Institute (AFBI) Animal Ethics Committee, Northern Ireland, UK and carried out in accordance with the conditions of the operating license issued from the Department of Health, Social Services and Public UK by the Animal (Scientific Procedures) Act 1986 (License No. PPL 2771). All animal work was performed and reported according to the ARRIVE guidelines.

### Preparation of murine macrophages

Peritoneal macrophages were separated from the complete exudate cell population by negative selection using magnetic beads in accordance with the manufacturer’s instructions (Miltenyi, USA). Bone marrow cells isolated from BALB/c mice were differentiated for 6 days in RPMI 1640 supplemented with FBS (10%v/v) and recombinant M-CSF (50 ng/ml; eBioscience, San Diego, CA, USA).

### Small RNA library preparation and bioinformatics analysis

The quality of the raw fastq files was assessed using FastQC and the adapters were removed using Cutadapt v1.13. The sequences with length smaller that 18 bp were discarded also using Cutadapt. The reads were then pre-processed with an in-house perl script that filters homopolymers, sequences that contain Ns and low quality reads. The mature miRNA sequences of Mus musculus were retrieved from miRbase and used to build the index for the alignment using bowtie. After the pre-processing, the reads were aligned against the mouse mature miRNAs. The reads that did not align against the mouse mature miRNAs were aligned against the *F. hepatica* known miRNAs. After the alignments, another in-house script was used to count the reads that were successfully mapped.

### Preparation of parasite RNA

*Fasciola hepatica* metacercariae were sourced from Ridgeway Research Ltd, UK. The South Gloucester isolate was used to infect sheep which allowed the recovery of adult worms from the bile ducts. BALB/c mice aged 6–8 weeks were orally infected with 30 metacercariae of *F. hepatica* (Italian isolate, Ridgeway Research Ltd) to recover the 21 day old immature flukes. All animal experimental procedures were carried out at Queen’s University Belfast, UK under license from the Home Office by the Animal (Scientific Procedures) Act 1986 (License No. PPL/2806) after ethical review by the Queen’s University Belfast Animal Welfare and Ethical Review Body. The Italian isolate was also used for the excystment of newly excysted juveniles (NEJ)^49^ and parasite culture for the recovery of extracellular vesicles (EVs)^50^. Total RNA from all parasites stages (NEJ, NEJ EV, immature and adult worms) was extracted using the miRNeasy Mini Kit (Qiagen) according to the manufacturer’s instructions, eluted in 30 μl RNase-free water.

*Caenorhabditis elegans* were maintained in 35 mm diameter petri dishes containing Nematode Growth Medium agar, (3 g NaCl, 17 g agar, 2.5 g peptone, 1 mL 1 M CaCl_2_, 1 mL 5 mg/mL cholesterol in ethanol, 1 ml 1 M MgSO_4_, 25 ml 1 M KPO_4_ buffer [pH 6.0, 108.3 g KH_2_PO_4_, 35.6 g K_2_HPO_4_, H_2_O to 1 L]) seeded with a lawn of *Escherichia coli* OP50. After 3–4 days, when all life stages of worms were present (embryo, larvae stages 1–4, and adult), worms were sub-cultured into new plates containing NGM agar and *E. coli* OP50. Once adequate numbers were achieved, worms were harvested by washing petri dishes with S buffer (129 mL 0.05 M KH_2_PO_4_, 871 mL 0.05 M KH_2_PO_4_, and 5.85 g NaCl), and scraped off agar with a sterile glass rod. The worm suspension was then centrifuged at 1000×*g* for 5 min, and the pellet containing the worms washed with sterile PBS. The final worm pellet was resuspended in 1 mL RNAzol RT (Molecular Research Centre Inc, USA) and homogenised with mortar and pestle for RNA extraction.

Adult males and female *S. mansoni* worms (n = 166) in RNAlater stabilisation solution (Life Technologies, USA) was kindly donated by Prof. Donald McManus (Queensland Institute of Medical Research). Worms were washed with sterile PBS to remove RNAlater by centrifugation at 1000×*g* for 5 min at 4 °C. The final worm pellet was resuspended in 2 mL of RNAzol RT and homogenised with mortar and pestle for RNA extraction.

### Preparation of macrophage extracellular vesicles

RAW 264.7 macrophages were cultured to confluency in RPMI + 10% FBS (v/v) at 37 °C and 5% CO_2_. Cells were collected by scraping and cellular debris removed by sequential centrifugation steps of 2000×*g* for 20 min at 4 °C followed by 10,000×*g* for 30 min at 4 °C. To obtain the extracellular vesicles the supernatant was subjected to ultracentrifugation at 104,492×*g* for 2 h at 4 °C and the pelleted material re-suspended to maximum volume depending on the size of the centrifuge tube. This resuspension was then ultracentrifuged at 104,492×*g* overnight at 4 °C to separate the soluble proteins (now in the supernatant) and vesicles (now in the pellet).

### miRNA target prediction

To determine possible gene targets of selected *F. hepatica* miRNA, the miRNA sequence of *fhe-miR-125b* was initially analysed using custom prediction with a murine background on the online miRNA target prediction tool, miRDB (mirdb.org). Additionally, mouse mRNA 3′UTR sequences were analysed for *fhe-miR-125b* target pairing using miRNA target predictive tools miRanda (http://www.microrna.org/) and TargetScan (http://www.targetscan.org). miRNA:mRNA pairings with a target score of > 60 were considered in miRDB, target score > 155 and minimum free energy (MFE) < − 20 (Energy-Kcal/Mol) were considered in miRanda, and a seed sequence match of 7mer-m8 site in TargetScan. Only common genes determined by all predictive tools were considered for further analysis. The acquired list of gene targets was analysed using the Database for Annotation, Visualization and Integrated Discovery (DAVID) v6.7 (david.ncifcrf.gov) and significant pathways targeted by fhe-miR-125a were determined using the murine Kyoto Encyclopaedia of Genes and Genomes (KEGG) database^39^.

### Microarray analysis

Microarray analyses were performed using the Illumina whole genome microarray platform (Illumina, USA) as previously described^51^. Quality control of data was performed using GenomeStudio (version 1.1.1, Illumina) by examining intensity histograms of hybridisation efficiency and noise. GeneSpring GX version 11 (Agilent Technologies, Foster City, USA) was used for all subsequent data analyses. All data were entered into GeneSpring GX 11 and normalised to the 75th percentile. Data for each replicate were normalised to respective uninfected controls and filtered for significance on the basis of detection score, a GenomeStudio generated measure of signal intensity relative to background. At least half of all hybridisations had to have a detection score > 0.949, (which equates to a confidence value of p ≤ 0.05) for a gene to be accepted. Gene expression values for commonly up- and down-regulated genes were averaged and a Students t-test was performed to identify significant changes in gene expression compared with controls (p ≤ 0.05). An additional cut-off of ± twofold change in expression was applied to identify changes in gene expression with likely biological significance.

### Transfection of bone marrow derived murine macrophages

Bone marrow derived macrophages (1 × 10^6^ cells/500 µl) were transfected with synthetic miRNAs (20 nM; (IDT, USA) combined with Lipofectamine RNAiMAX (Life Technologies, USA) over a period of 24 h in accordance to manufacturer’s protocol. Transfected cells were washed with sterile PBS before stimulation with *E. coli* lipopolysaccharide (LPS; 10 ng/ml) for 6 h at 37 °C and 5% CO_2_ to induce an inflammatory response. Non-transfected cells, and cells treated with Lipofectamine RNAiMAX alone, without LPS stimulation, were also analysed to control for contamination and possible immune responses induced by transfection reagents. Non-transfected cells were stimulated with LPS to determine the baseline inflammatory response of cultured macrophages. After LPS stimulation, the culture media was collected from all treatment groups for cytokine assays, and the cells washed with sterile PBS and collected for RNA extraction.

### RNA extraction from mammalian cells

Cells or extracellular vesicles were homogenised with the RNAzol RT at 1 mL to 10^7^ cells. Ultrapure DNAse/RNase free distilled water (dH_2_O) (Life Technologies, USA) was added to the homogenate, at 0.4 mL of dH_2_O per 1 mL of RNAzol RT used, and then centrifuged at 12,000×*g* for 15 min at 4 °C, to pellet DNA, proteins, and polysaccharides. Supernatant containing soluble RNA was collected and combined with 4-bromoanisole (BAN; Sigma-Aldrich, USA) at 0.5% of supernatant volume, and then centrifuged at 12,000×*g* for 10 min at 4 °C to pellet residual DNA, proteins, and polysaccharides. To precipitate RNA, the supernatant was collected and combined with isopropanol (100% v/v; Sigma-Aldrich, USA) and 5 μL glycogen (5 mg/mL; Life Technologies, USA), and incubated overnight at − 20 °C. The RNA precipitate was pelleted by centrifugation at 12,000×*g* for 10 min at 4 °C, washed with 500 µL ethanol (75% v/v; Sigma-Aldrich, USA) twice and re-solubilised in 25 µL dH_2_O. Concentrations and quality of isolated RNA was measured using the NanoDrop One/One UV–Vis spectrophotometer (Life Technologies).

### Pull-down of argonaute-2 (Ago-2)-bound miRNA from peritoneal macrophages

Peritoneal macrophages were collected and pooled from five uninfected or infected mice for Ago2 pulldown using the MagCapture microRNA isolation kit specific for mouse Ago-2 (FUJIFILM Wako Pure Chemical, JPN) according to manufacturer’s instructions. Briefly, cleared cell lysate from each pooled sample of 2 × 10^6^ macrophages was incubated with AntiMouse Ago2 magnetic beads for 2 h at 4 °C with rotation. The beads were then washed and subjected to Proteinase K digestion. RNA was isolated from the digest using RNAzol RT and quantified using Qubit Fluorometer with a microRNA Assay kit (ThermoFisher Scientific). The miRNA samples were then normalised to a concentration of 0.5 ng/sample for RT-qPCR. This two-step normalisation of cell number and input miRNA ensured that all samples contained similar amounts of miRNA for comparable analysis by RT-qPCR.

### RT-qPCR

After extraction of RNA, all samples were normalised to 100 ng/μL prior to cDNA synthesis. cDNA synthesis was carried out using the TaqMan MicroRNA Reverse Transcription Kit (Life Technologies, USA) according to manufacturer’s instructions. Synthesised cDNA was further diluted 1:4 dH_2_O. For RT-qPCR, 1 µL of diluted cDNA was combined with 5 µL of SensiFAST Probe Hi-ROX Kit (Bioline, UK), 3.5 µL dH_2_O, and 0.5 µL of TaqMan Probe for gene expression or miRNA. Samples were prepared on a MicroAmp optical 96-well reaction plate as technical triplicates (Life Technologies, USA) and analysed using the QuantStudio 6 flex real-time PCR system (Life Technologies, USA). Data was analysed using LinRegPCR (v2017.1), and presented as absolute expression of genetic material prior to amplification (N0). The N0 value is the raw fluorescent value and an indication of the expression.

### Cytokine assay

The quantities of IL-6 and TNF secreted by transfected bone marrow derived macrophages was determined using ELISA kits (BD Biosciences, USA) according to manufacturer’s instructions.

### Statistical analysis

Results were analysed using Graphpad prism software (version 8). Comparisons between samples were made using one-way ANOVA with Tukey post hoc test. Differences were considered significant when *P* ≤ 0.05.

## Supplementary Information

Supplementary Information.

Supplementary Table S1.

Supplementary Table S2.

Supplementary Table S3.
