# Supplementary material for: Fasciola hepatica hijacks host macrophage miRNA machinery to modulate early innate immune responses
Source: Sci Rep. 2021 Mar 24;11:6712. doi: 10.1038/s41598-021-86125-1 (PMC7990952; doi:10.1038/s41598-021-86125-1)
Supplement: Supplementary file 1 — Supplementary Information. [file 41598_2021_86125_MOESM1_ESM.docx]

***Fasciola hepatica* hijacks host macrophage miRNA machinery to modulate early innate immune responses**

Nham Tran^1†^, Alison Ricafrente^2†^, Joyce To^2^, Maria Lund^2^, Tania M. Marques^3^, Margarida Gama-Carvalho^3^, Krystyna Cwiklinski^4^, John P. Dalton^4^, Sheila Donnelly^2*^

^1^School of Biomedical Engineering, Faculty of Engineering and Information Technology, The University of Technology Sydney, Ultimo, NSW, Australia.

^2^School of Life Sciences, Faculty of Science, The University of Technology Sydney, Ultimo, NSW, Australia.

^3^ BioISI – Biosystems & Integrative Sciences Institute, Faculty of Sciences, University of Lisbon, 1749-016, Lisboa, Portugal

^4^ Center of One Health (COH) and Ryan Institute, School of Natural Science, National University of Ireland Galway, Galway, Ireland.

*Correspondence to: Sheila.Donnelly@uts.edu.au

†These authors contributed equally to the work

**Supplementary Figure 1:** GO analysis of pathways represented by the genes that were significantly (p>0.05) downregulated (>2 fold change in expression) in macrophages harvested from the peritoneal cavity of mice infected with *F. hepatica.* The image was created at https://www.bioconductor.org

**Supplementary Figure 2:** The Argonaute pull down protocol was optimised using RAW macrophages (10 x 10^6^ cells) and the presence of Ago-2 within the harvested products confirmed by immunoblot using a murine specific Anti-Ago-2 antibody

**Supplementary Figure 3:** Interactome showing the names for each node and their interacting partners. Traf6 is marked by a red dotted square, with all pathway associations shown in blue. The Illustration was created at https://www.innatedb.com.

**Supplementary Table legends:**

**Supplementary Table 1:** Putative gene targets for mature *fhe-miR-125b*, generated using the online miRNA target prediction tools miRanda, TargetScan and miRDB.

**Supplementary Table 2:** The top ten KEGG pathways generated from gene ontology analysis of the predicted gene target list conducted against a *Mus musculus* background using GO tool DAVID.

**Supplementary Table 3:** Downregulated (>2 fold change in expression; p>0.05) genes within the peritoneal macrophage of mice 18h after an oral infection with *F. hepatica* as determined by the Illumina whole genome microarray platform.
